# Light Sheet Microscopy Using FITC-Albumin Followed by Immunohistochemistry of the Same Rehydrated Brains Reveals Ischemic Brain Injury and Early Microvascular Remodeling

**DOI:** 10.3389/fncel.2020.625513

**Published:** 2021-01-05

**Authors:** Ayan Mohamud Yusuf, Nina Hagemann, Sarah Schulten, Olessja Rausch, Kristina Wagner, Tanja Hussner, Yachao Qi, Matthias Totzeck, Christoph Kleinschnitz, Anthony Squire, Matthias Gunzer, Dirk M. Hermann

**Affiliations:** ^1^Department of Neurology, University Hospital Essen, University Duisburg-Essen, Essen, Germany; ^2^Department of Cardiology, University Hospital Essen, University Duisburg-Essen, Essen, Germany; ^3^Institute for Experimental Immunology and Imaging, University Hospital Essen, University Duisburg-Essen, Essen, Germany; ^4^Leibniz Institute for Analytical Sciences ISAS e.V., Dortmund, Germany

**Keywords:** angiography, brain clearing, capillary, cerebral microvessels, focal cerebral ischemia—reperfusion, ischemic stroke, microvascular remodeling

## Abstract

Until recently, the visualization of cerebral microvessels was hampered by the fact that only short segments of vessels could be evaluated in brain sections by histochemistry. These limitations have been overcome by light sheet microscopy, which allows the 3D analysis of microvasculature in cleared brains. A major limitation of light sheet microscopy is that antibodies do not sufficiently penetrate cleared brains. We herein describe a technique of reverse clearing and rehydration, which after microvascular network analysis allows brain sectioning and immunohistochemistry employing a broad set of antibodies. Performing light sheet microscopy on brains of mice exposed to intraluminal middle cerebral artery occlusion (MCAO), we show that in the early phase of microvascular remodeling branching point density was markedly reduced, more strongly than microvascular length. Brain infarcts in light sheet microscopy were sharply demarcated by their autofluorescence signal, closely corresponding to brain infarcts revealed by Nissl staining. Neuronal survival, leukocyte infiltration, and astrocytic reactivity could be evaluated by immunohistochemistry in rehydrated brains, as shown in direct comparisons with non-cleared brains. Immunohistochemistry revealed microthrombi in ischemic microvessels that were likely responsible for the marked branching point loss. The balance between microvascular thrombosis and remodeling warrants further studies at later time-points after stroke.

## Introduction

The analysis of vascular networks and their responses to ischemic injury is crucial for the understanding of stroke pathogenesis (Hermann and Zechariah, 2009; Grutzendler and Nedergaard, 2019). Following focal cerebral ischemia, microvessels degenerate in more severely affected brain areas (Del Zoppo et al., 1991; Göb et al., 2015). In the subsequent stroke recovery phase, the degree of successful microvascular network remodeling defines the extent of brain plasticity, and neurological recovery (Hermann and Chopp, 2012). The visualization of cerebral microvessels was hampered until recently by the fact that only short segments of microvessels could be evaluated in 2D brain sections by histochemistry. 3D reconstruction of vasculature networks from these required a large number of high-quality consecutive brain sections (Boero et al., 1999; Tsai et al., 2009), making this methodology impractical for larger-scale evaluations of vascular remodeling. These limitations have recently been overcome by light sheet microscopy in brains of ischemic mice, to which a gelatin hydrogel containing fluorescein isothiocyanate (FITC) -conjugated albumin had transcardially been administered immediately before sacrifice (Lugo-Hernandez et al., 2017). Following solvent-based tissue clearing, image acquisition, segmentation, and rendering, microvascular networks can be analyzed in an automated way by the fitting of the blood vessels to a filament model (Lugo-Hernandez et al., 2017). This strategy has meanwhile been adopted by others and us using anti-CD31 or wheat germ agglutinin (WGA) for microangiography (Merz et al., 2019; Kirst et al., 2020; Todorov et al., 2020). A major drawback of light sheet microscopy is that antibodies poorly penetrate the cleared brains and that cleared brains cannot be cut on a cryostat or vibratome because the texture of the tissue is too firm to allow brain sectioning. As such, microvascular light sheet microscopy studies and immunohistochemistry could not yet be performed in the same animals. To overcome this shortcoming, we herein developed a protocol allowing rehydration of cleared brains, followed by histochemical brain sectioning and immunohistochemistry. Using an advanced analysis strategy, we evaluated microvascular network characteristics, showing that the early phase of microvascular remodeling after intraluminal middle cerebral artery (MCA) occlusion is characterized by the preferential loss of branching points that exceeds the loss of microvascular length. Using immunohistochemistry, microthrombi were identified in ischemic microvessels that were likely responsible for marked branching point loss.

## Materials and Methods

### Animals

Animal experiments were performed following the regulations of the National Institute of Health Guidelines for the Care and Use of Laboratory Animals in compliance with ARRIVE guidelines and the permission of local authorities (Landesamt für Natur, Umwelt und Verbraucherschutz, North-Rhine Westphalia). Male C57Bl/6j mice (25–30 g body weight, 10–12 weeks; Envigo, Horst, Netherlands) were kept in a 12 h-12 h light/dark cycle with free access to food and water in groups of five animals per cage. For the optimization of clearing procedures, sets of healthy mice were used. In a subsequent step, mice exposed to focal cerebral ischemia were evaluated. As exclusion criteria, mice were removed from the study when suffering from respiratory abnormalities or severe motor handicaps and swallowing problems resulting in a weight loss >20%.

### Focal Cerebral Ischemia

Middle cerebral artery occlusion (MCAO) was performed using an intraluminal filament technique (Wang et al., 2020) as previously reported. Briefly, male C57Bl/6j mice were anesthetized using 1% isoflurane (30% O_2_ and remainder N_2_O), while body temperature was maintained between 36.5 and 37.0°C using a feedback heating system (Fluovac, Harvard Apparatus, Holliston, MA, USA). An incision at the neck midline was performed to dissect the left common and external carotid arteries. The common carotid artery was sutured and the internal carotid artery was transiently clipped. To occlude the MCA, a silicon resin-coated nylon monofilament was introduced through a small cut in the left common carotid and advanced to the left internal carotid artery until reaching the origin of the left MCA at the circle of Willis. MCAO lasted 30 min. Blood supply was reestablished by the withdrawal of the monofilament. Laser Doppler flow was monitored during ischemia and up to 20 min after reperfusion using a flexible 0.5 mm fiber-optic probe (Perimed, Rommerskirchen, Germany) attached to the intact skull overlying the MCA territory (2 mm posterior, 6 mm lateral from Bregma). After the surgery, wounds were sealed, the anesthesia was discontinued and animals were placed in a warming cabinet (37.0°C) for 1 h to recover. Analgesia was ensured by subcutaneous injection of 0.1 mg/kg buprenorphine (Temgesic; Essex Pharma, Munich, Germany) before surgery and subcutaneous injection of 4 mg/kg carprofen (Bayer Vital, Leverkusen, Germany) directly after MCAO and thereafter daily for 3 days at 24-h-intervals.

### Hydrogel Preparation

The accurate quantification of microvessels requires a fluorescent dye solution with low viscosity for efficient penetration of microvessels, that would retain a strong fluorescent signal with the use of organic solvents, and would not result in excessively high background fluorescence in the sample due to escape from damaged blood vessels. This technical challenge was solved by the use of a gel as reported previously (Lugo-Hernandez et al., 2017) with slight modifications. Briefly, a solution of 2% (w/v) gelatin (Sigma-Aldrich, Deisenhofen, Germany) was prepared in phosphate-buffered saline (PBS; PBS tablets, Merck-Millipore, Darmstadt, Germany) at 60°C and allowed to cool down to 40°C with constant stirring. Then, FITC conjugated albumin (Sigma-Aldrich) was added to the gelatin solution at a concentration of 0.1% (w/v). The gel was filtered using filter paper (GE Whatman, Dassel, Germany) and continuously stirred at 30°C to avoid excessive evaporation.

### Animal Sacrifice and Hydrogel Perfusion

Seven days post-MCAO, mice were deeply anesthetized and transcardially perfused with 40 ml of PBS containing 50 U/ml heparin (Ratiopharm, Ulm, Germany), followed by perfusion of 20 ml of 4% paraformaldehyde (PFA; Merck-Millipore) in PBS. Ten microliter of hydrogel was subsequently perfused and the mouse bodies were then placed head down into ice water over 15 min for solidification of the hydrogel. The brains were carefully removed and incubated in 4% PFA in PBS at 4°C overnight.

### Whole Brain Clearing

For clearing adult mouse brains perfused with hydrogel we adapted the 3DISCO clearing technique (Lugo-Hernandez et al., 2017; Kirst et al., 2020), which combines the use of two organic solvents, i.e., tetrahydrofuran (THF; Sigma–Aldrich) for dehydration and lipid solvation and ethyl cinnamate (ECi; Sigma–Aldrich) for matching the refractive index of the remaining dehydrated sample. Incubation of brains in THF was performed for 12 h each in increasing concentrations (30%, 60%, 80%, and 100%) at room temperature with constant agitation at 300 rpm using a horizontal shaker under a laminar flow hood. To ensure complete dehydration, samples were immersed in solutions of the last THF gradient (100%) twice. Then, samples were incubated in ECi for 12 h with continued agitation and stored in this solvent until image acquisition. All incubation steps were done in 30 ml of each solvent in dark brown glass bottles.

### Reverse Clearing and Rehydration

Dehydration and clearing of brains lead to tissue hardening. Cleared brains cannot be cut on cryostats or vibratomes. To overcome this limitation, we established a rehydration protocol of cleared brains using the same THF concentrations used for dehydration but in reversed order. Briefly, two incubation steps using 100% THF for 12 h were performed before brains were immersed in 80%, 60%, and 30% THF. Subsequently, brains were incubated in 15% (w/v) sucrose for 6 h at 4°C followed by 30% sucrose for 48 h. The latter step was indispensable for enabling brain freezing and cutting. Brains were frozen using dry ice and stored at −20°C until tissue sectioning. Twenty micrometer coronal sections were obtained from PFA fixed and rehydrated brains on a cryostat.

### Light Sheet Data Acquisition and Image Processing

Cleared whole mouse brains were imaged using a commercial light sheet Ultramicroscope 2 (LaVision BioTec, Bielefeld, Germany) that is based on an Olympus MVX10 zoom microscope which, when combined with a 2× 0.5 NA objective, gave a magnification range from 1.26 to 12.6. The Ultramicroscope was equipped with bidirectional light sheet illumination, and an Andor Neo sCMOS camera having a 2,560 × 2,160 chip of 6.5 μm pixel size. We performed serial optical imaging of the brains in a ventral-dorsal direction by exciting the FITC-albumin labeled vessels using a 488 nm diode laser and a 525/50 nm band-pass emission filter. Autofluorescence was captured with a 561 nm laser and detected *via* a 595/40 nm band-pass emission filter. Images of the respective brain hemisphere were acquired at 6.4× magnification with 2 μm steps in the axial direction using the dynamic focus and the highest NA of the light sheet illumination for optimal axial resolution. For image rendering of the brains, Bitplane software (Imaris, Cologne, Germany) was used.

### Vascular Quantification by Light Sheet Microscopy

For detailed vascular quantification in stroke brains, two image stacks were taken in corresponding ischemic (ipsilateral) and non-ischemic (contralateral) areas within the lateral-caudal portion of the striatum, which was acquired with 6.4× magnification and a step size of 2 μm in ventrodorsal direction, starting at Bregma −6.24 mm^1^ and covering a distance of 1 mm which results in the acquisition of 501 images. From each of these image stacks, regions of interest (ROI) measuring 500 × 500 × 1000 μm were chosen at the dorsolateral pole of the striatum at the border of the external capsule. In these ROIs, microvascular length density and branching point density was measured after network modeling using the Imaris 3D rendering software filament tracer tool. As a consequence of the much improved axial resolution, the microvascular network characteristics reported here, exceed those reported in the earlier study (Lugo-Hernandez et al., 2017).

### Analysis of Infarct and Edema Size by Light Sheet Microscopy

In the light sheet images, brain infarcts were sharply demarcated from the surrounding tissue based on autofluorescence signal at a wavelength of 561 nm that was elevated in infarcted tissue. To determine, whether the autofluorescence signal indeed corresponded to brain infarcts and brain edema, we measured their volume and area in Imaris and corrected it for brain shrinkage.

### Light Sheet Image Preprocessing and Data Analysis

For the detailed vascular analysis image stacks were preprocessed using open source software ImageJ (National Institutes of Health, Bethesda, MD, USA) and python scripts from the Vascular Modelling Toolkit (VMTK^2^). In ImageJ image stacks were first Gaussian smoothed (Sigma = 2 μm) before performing a rolling ball background subtraction (radius = 20 μm). A vessel enhancement was then applied using a multiscale Frangi vesselness filter (Frangi et al., 1998).

### Histochemical Brain Infarct Analysis

Twenty micrometer coronal brain sections of PFA fixed brains or PFA-fixed rehydrated brains collected at 1 mm intervals were stained with cresyl violet. In these sections, the border between infarcted and non-infarcted tissue was outlined using ImageJ [National Institutes of Health (NIH), Bethesda, MD, USA]. Infarct volume and area were determined by subtracting the area of the non-lesioned ipsilateral hemisphere from the area of the contralateral hemisphere and, in the case of volume, integrating areas across the brain (Wang et al., 2018). Brain edema was calculated as the volume or area difference of the ipsilateral and the contralateral hemisphere (Wang et al., 2018). Effects of brain length shrinkage due to dehydration and clearing or brain length expansion due to reverse clearing and rehydration were adjusted for in all analyses.

### Immunohistochemistry

Sections of rehydrated brains were dried at 37°C for 30 min. For antigen retrieval, sections were then immersed in 0.01 M citrate buffer (pH 5.0) that was heated in a microwave oven for 15 min. Sections were postfixed with ice-cold acetone/methanol for 3 min and washed three times with 0.1 M PBS containing 0.2% Tween-20 for 5 min each. Brain sections were blocked using 0.1 M PBS containing 0.2% Tween-20, 5% normal donkey serum, and 1% BSA for 30 min in a humid chamber. Thereafter, samples were incubated with primary antibodies overnight at 4°C followed by three washing steps using 0.1 M PBS/0.2% Tween-20 and secondary antibody incubation for 1 h at room temperature. The following primary antibodies were used: mouse anti-NeuN (clone 13E6, Millipore), rat anti-GFAP (clone 2.2B10, Invitrogen), rat anti-CD45 (clone 30-F11, BD Biosciences), rat anti-CD31 (clone MEC 13.3, BD Biosciences), rabbit anti-collagen type-IV (AB756P, Millipore) and rat anti-GPIbα (clone Xia.B2, Emfret Analytics). Samples were labeled with secondary donkey Alexa Fluor 594 conjugated anti-mouse, donkey anti-rat, donkey anti-rabbit, or biotinylated antibody (Invitrogen). Sections stained with biotinylated antibody were revealed by 3,3′-diaminobenzidine (DAB) staining using an avidin-biotin complex (ABC) peroxidase kit (Vectastain Elite Kit Standard, Vector Laboratories). In sections stained with fluorescent antibodies, nuclei were counterstained with Hoechst33342 solution (Sigma-Aldrich).

### Image Acquisition of Immunohistochemical Stainings From PFA-Fixed and PFA-Fixed Rehydrated Brains

Images covering the ischemic or non-ischemic hemisphere were acquired using a Zeiss AxioObserver.Z1 Inverted Microscope at 20× magnification. Tiled images were merged using ZEN blue software (Zeiss).

### Statistical Analysis

All data are presented as means ± SD values. To determine differences in vessel density between ischemic and non-ischemic brain areas, paired Student’s *t*-tests were used. For comparisons between ≥3 groups, we employed one-way analysis of variance (ANOVA) tests followed by Tukey’s *post hoc* tests. *P-*values of ≤0.05 were considered significant. Statistical analysis was performed using Graphpad Prism 7.0.

## Results

### Changes in Brain Length During Dehydration and Rehydration

**Figure 1:**
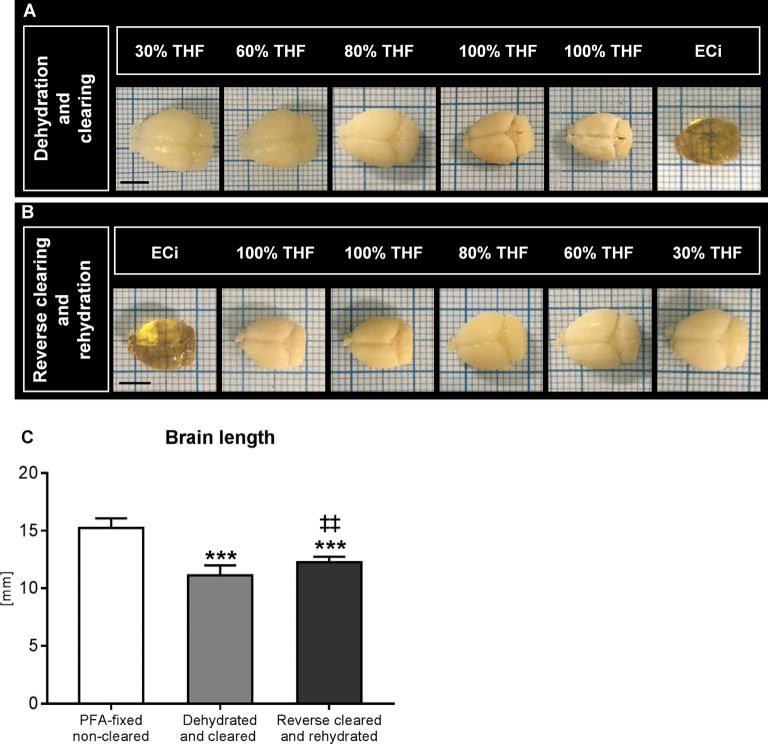
Analysis of brain length after dehydration and clearing and after reverse clearing and rehydration. Representative brains exposed to **(A)** dehydration and clearing using increasing tetrahydrofuran (THF) concentrations and ethyl cinnamate (ECi) and **(B)** reverse clearing and rehydration using decreasing THF concentrations in front of millimeter scales. A quantitative analysis of the rostrocaudal length of paraformaldehyde (PFA)-fixed brains, brains submitted to dehydration and clearing, and brains submitted to reverse clearing and rehydration is presented in **(C)**. Data are means ± SD values. ***p ≤ 0.001 compared with PFA-fixed native brains; ^‡‡^*p* ≤ 0.01 compared with dehydrated and cleared brains, *n* = 4–9 animals/group [in **(C)**]. Scale bars, 5 mm [in **(A,B)**].

During dehydration and optical clearing with the modified 3DISCO procedure (Lugo-Hernandez et al., 2017; Merz et al., 2019) repeated photographs of the brains were taken, which revealed that brain rostrocaudal length decreased by 27.0% ± 5.7% during the dehydration and clearing process (Figure 1A). Brain shrinkage was partly reversed by reverse clearing and rehydration (Figure 1B), but the rostrocaudal length of rehydrated brains was still 19.4% ± 3.0% below that of PFA-fixed native brains before clearing (Figure 1C).

### Visualization of Brain Infarcts Using Light Sheet Microscopy

**Figure 2:**
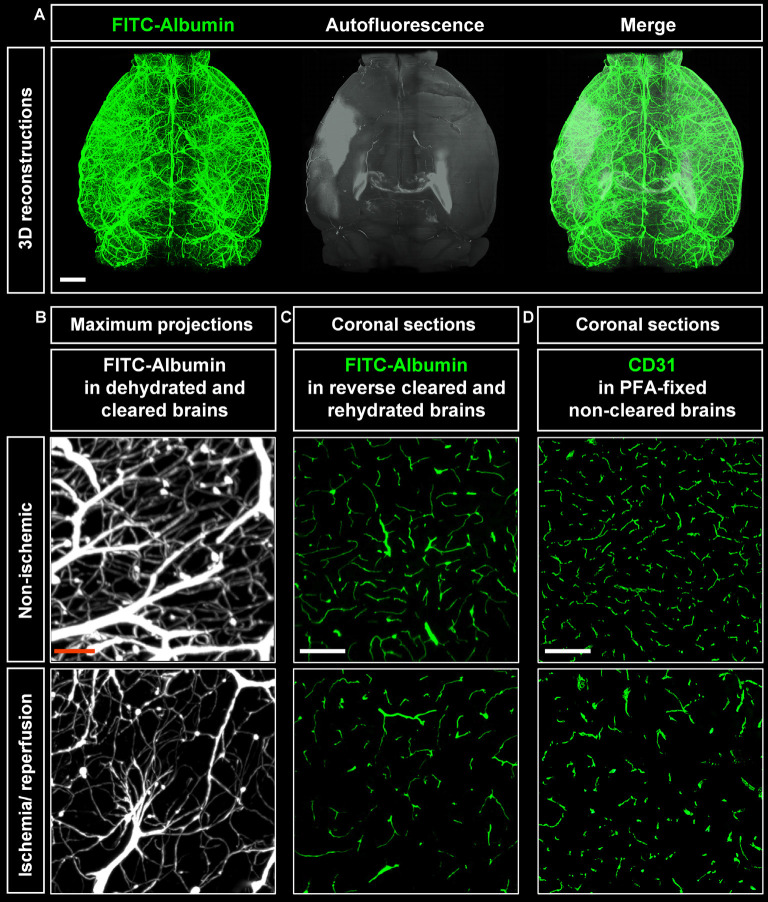
Visualization of vascular networks using light sheet fluorescence microscopy (LSFM). **(A)** Representative 3D reconstructions of ischemic brains from C57BL/6j wildtype mice exposed to transient middle cerebral artery occlusion (MCAO) depicting FITC-albumin labeled microvessels (green) and the autofluorescence signal (gray). **(B)** Maximum projection image stacks (100 μm in dorso-ventral direction) of FITC-albumin labeled microvessels in the reperfused ischemic striatum and the contralateral non-ischemic striatum of C57BL/6j mice exposed to transient MCAO, followed by animal sacrifice after 7 days. **(C)** FITC-albumin labeled microvessels or **(D)** CD31 stained microvessels in coronal brain sections obtained from the reperfused ischemic striatum and the contralateral non-ischemic striatum of C57BL/6j mice exposed to transient MCAO that were sacrificed after 7 days. Scale bars, 1 mm [in **(A)**]; 50 μm [in **(B)**]; 100 μm [in **(C,D)**].

Cleared whole brain specimens were scanned using the light sheet microscope with 1.6× magnification. 3D reconstructions of autofluorescence signals excited at 561 nm and FITC-albumin labeled microvessels excited at 488 nm were used to visualize the brain topology (Figure 2A). Interestingly, infarcted tissue was demarcated by its autofluorescence signal at 561 nm excitation (Figure 2A). Notably, the infarct extension revealed by this autofluorescence signal very closely resembled that in cresyl violet stainings. A representative coronal autofluorescence image depicting a typical brain infarct at the rostrocaudal level of the striatum as opposed to a representative cresyl violet staining depicting the same infarct at the same rostrocaudal level is shown in Figure 4A.

**Figure 3:**
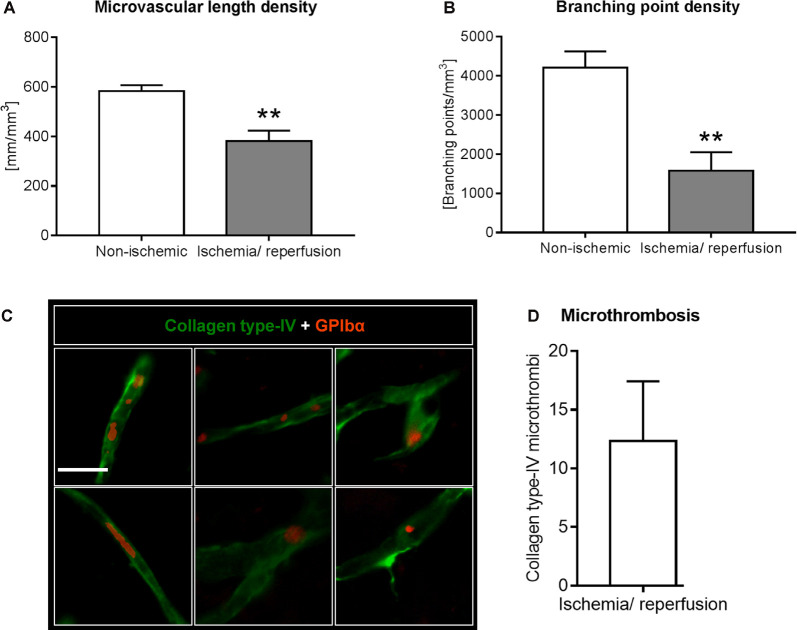
Analysis of microvascular network characteristics by LSFM after ischemia/ reperfusion. Microvascular network characteristics were analyzed with the filament tracer model of the Imaris 3D software package. **(A)** Microvascular length density and **(B)** branching point density evaluated in the reperfused ischemic striatum and the contralateral non-ischemic striatum of C57BL/6j mice exposed to transient MCAO that were sacrificed after 7 days. **(C,D)** Cerebral micro-thrombosis evaluated by the number of glycoproteins (GP)-Ibα (GPIbα)^+^ platelet aggregates in collagen type-IV^+^ microvessels in the ischemic striatum of C57BL/6j mice exposed to transient MCAO that were sacrificed after 7 days. Data are means ± SD values. **p ≤ 0.01 compared with contralateral non-ischemic striatum **(C)**; *n* = 3 animals/group [in **(A,B)**]; *n* = 6 animals/ group [in **(D)**]. Scale bar, 20 μm [in **(C)**].

**Figure 4:**
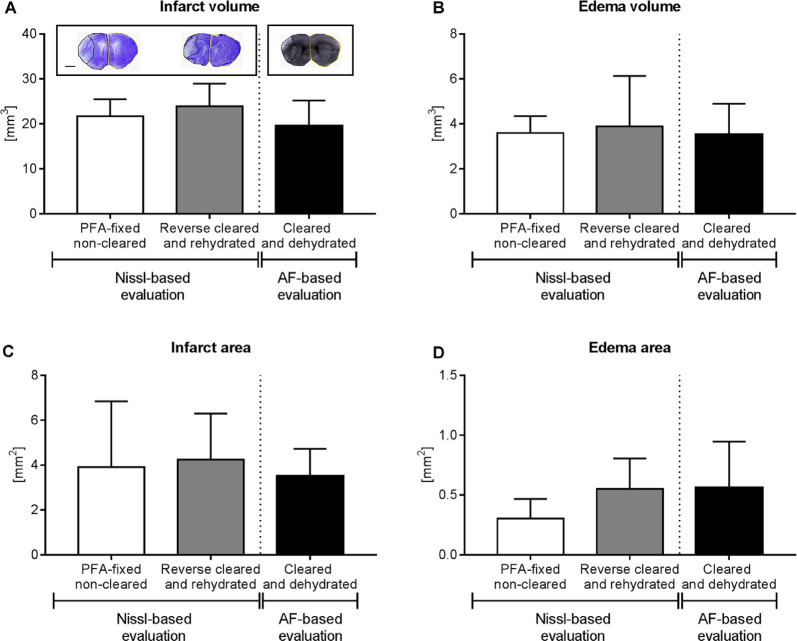
Brain infarct analysis in PFA-fixed non-cleared brains and brains exposed to reverse clearing and rehydration. **(A)** Infarct volume, **(B)** edema volume, **(C)** infarct area, and **(D)** edema area evaluated on cresyl violet (Nissl)-stained coronal brain sections from PFA-fixed non-cleared brains and PFA-fixed brains subjected to a clearing, reverse clearing and rehydration and on brain autofluorescent (AF) signal of PFA-fixed cleared brains evaluated by LSFM. C57BL/6j mice had been exposed to transient MCAO, followed by animal sacrifice after 7 days. Brain shrinkage in the different conditions was adjusted for. Representative coronal brain sections are shown **(A)**. Data are means ± SD values, *n* = 6–8 animals per group [in **(A–D)**]. Scale bar, 1,000 μm [in **(A)**].

### Visualization of Vascular Networks Using Light Sheet Microscopy

Maximum projection image stacks covering a thickness of 100 μm in the dorso-ventral direction revealed the loss of capillaries upon ischemia/ reperfusion (I/R; Figure 2B). FITC-albumin vessel labeling persisted in coronal sections even after rehydration (Figure 2C) and could be used to study the co-localization of other markers with microvessels. Furthermore, immunofluorescent staining of sections from PFA-fixed brains with the endothelial marker CD31 closely resembled the fluorescent signal from FITC-albumin in rehydrated brain sections (Figure 2D), suggesting that histochemical staining of microvessels is dispensable in FITC-albumin hydrogel perfused sections for estimating microvascular density.

### Analysis of Microvascular Network Characteristics by Light Sheet Microscopy

Using the 3D light sheet image stacks, microvascular length density and branching point density were evaluated in regions of interest measuring 500 × 500 × 1000 μm in the ischemic and corresponding contralateral non-ischemic striatum. Microvascular length density and branching point density was reduced in the reperfused ischemic striatum (Figures 3A,B). Notably, the loss of branching points relative to the control situation (Figure 3B) was even more pronounced than the loss of microvascular length (Figure 3A).

### Analysis of Infarct Volume and Brain Edema in Rehydrated Brains

Because of the sequential brain shrinkage and volume expansion, we tested whether infarct size and brain edema could reliably be determined in ischemic brains that had undergone dehydration followed by clearing and rehydration. Thus, we compared the infarct volume, edema volume, infarct area, and edema area in coronal sections of PFA-fixed cleared and rehydrated brains with sections obtained from PFA-fixed native brains. The analysis of cresyl violet (Nissl) stainings showed that infarct volume (Figure 4A), edema volume (Figure 4B), infarct area (Figure 4C), and edema area (Figure 4D) were very similar in brains subjected to dehydration, clearing and rehydration and PFA-fixed non-cleared brains, when brain shrinkage was adjusted for using correction factors of 1.24^3^ (for analysis of infarct volume and edema volume) or 1.24^2^ (for analysis of infarct area and edema area). Moreover, Imaris analysis of the infarct volume, edema volume, infarct area, and edema area based on the LSFM-based brain autofluorescence signal revealed that the results were comparable to infarct and edema data obtained from cresyl violet (Nissl) stainings (Figures 4A–D) when brain shrinkage was adjusted for by using correction factors of 1.37^3^ (for analysis of infarct volume and edema volume) or 1.37^2^ (for analysis of infarct area and edema area). Hence, our protocol reveals a new reliable strategy for brain infarct analysis.

### Immunohistochemical Analysis of Rehydrated Brains Allows the Staining of a Wide Set of Parenchymal Brain Tissue Markers

**Figure 5:**
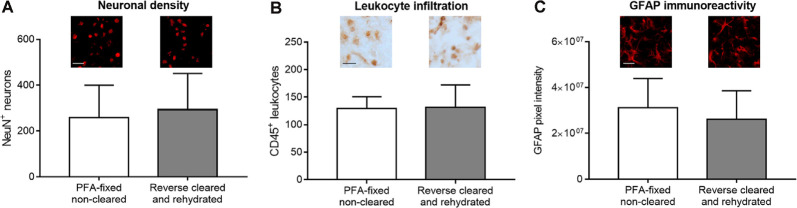
Comparison of surviving neurons, brain-invading leukocytes, and astroglial reactivity by immunohistochemistry in PFA-fixed non-cleared brains and brains exposed to reverse clearing and rehydration. **(A)** Number of NeuN^+^ neurons, **(B)** density of CD45^+^ brain-invading leukocytes, and **(C)** astrocytic GFAP immunoreactivity in the reperfused ischemic striatum evaluated on coronal sections at the bregma level obtained from PFA-fixed non-cleared brains and PFA-fixed brains subjected to clearing, reverse clearing, and rehydration. C57BL/6j mice had been exposed to transient MCAO, followed by animal sacrifice after 7 days. Brain shrinkage was adjusted for. Representative microphotographs are shown. Data are means ± SD values, *n* = 6–8 animals per group [in **(A–C)**]. Scale bars, 20 μm [in **(A–C)**].

In addition to brain infarct and edema measurements, the analysis of brain injury and remodeling includes a thorough investigation of parenchymal responses that comprises an analysis of surviving neurons, glial responses and immune cell infiltrates (Wang et al., 2020), besides others, for which immunohistochemistry is widely used. In our study, we labeled a set of widely used marker proteins using established immunohistochemistry protocols, again comparing brain sections from PFA-fixed cleared and rehydrated brains with PFA-fixed native brains. Notably, clearing and rehydration did not influence the number of NeuN^+^ neurons (Figure 5A), the density of CD45^+^ brain-invading leukocytes (Figure 5B) and GFAP pixel intensity as a marker for astrocytic reactivity (Figure 5C) in the ischemic striatum, which was evaluated as it represents the core of the MCA territory.

### The Immunohistochemical Analysis Identifies Microvascular Thrombosis as a Probable Mechanism Contributing to Altered Microvascular Network Characteristics

Based on the observation that the extent of branching point density reduction in the microvascular network analyses exceeded that of microvascular length reduction, we asked whether microvascular segments exhibited platelet aggregates, which predispose the brain for microvascular occlusions. Using immunostainings for the platelet marker GPIbα we examined the presence of platelet aggregates in previously ischemic microvessels. In the ischemic striatum, GPIbα^+^ platelet aggregates were noted in microvessels, indicative of micro thrombosis (Figures 3C,D). In the contralateral non-ischemic striatum, on the other hand, no platelet aggregates were found.

## Discussion

Herein, we have developed a powerful method to utilize high-resolution 3D light sheet microscopy in combination with conventional 2D immunohistochemistry. Using light sheet microscopy imaging of cleared brains in which vessels are fluorescently labeled, the brain microvascular network can be analyzed using an IMARIS-based filament model. Thus, microvascular network characteristics, such as microvascular length and branching point density, are extracted and allow for a comprehensive evaluation of microvascular features that goes beyond previous analyses. Thereby for the first time, we show that in the early stroke recovery phase, at 7 dpi, the reduction of branching points post-I/R exceeds that of microvascular length. This observation suggests that branching points were lost as a consequence of the occlusion of microvascular branches, resulting in the rarefication of microvascular networks. Immunohistochemical analyses revealed GPIb^+^ microthrombi in reperfused cerebral microvessels, providing a very probable explanation for the observed network changes. Following intraluminal MCAO, microvascular thrombosis was shown to contribute to ischemic brain injury via infiltrating T cells and macrophages (Göb et al., 2015; Schuhmann et al., 2017). Besides microvascular thrombosis, acute ischemic injury of microvessels may have added to the network changes noted.

Infarcted brain tissue emits a strong autofluorescence signal after intraluminal MCAO that can be visualized by light sheet microscopy and can be used as a landmark for the evaluation of microvascular responses. Herein, we demonstrate that the extension of this autofluorescence signal precisely corresponds to the brain infarct determined by cresyl violet staining. As such, the autofluorescence signal can now be used for the brain infarct and edema volumetry and planimetry, at least in the early post-acute time-window. Previous studies have already revealed that brain autofluorescence offers itself as a landmark to identify endogenous brain structures (Renier et al., 2016; Ye et al., 2016). Neither a comprehensive microvascular network analysis was performed post-I/R, nor ischemic injury was evaluated by LSFM in these earlier studies. The information obtained resembles that of brain infarcts and vascular networks in magnetic resonance imaging (MRI) and magnetic resonance angiography (MRA), with the important difference that microvessels can be traced at a microscopic capillary level. It is tempting to speculate that this new technique can help to bridge experimental and clinical stroke studies in the future.

The combination of LSFM with immunohistochemistry has hitherto not been possible in the reperfused ischemic brain. Immunohistochemistry of whole-brain specimens required huge amounts of primary and secondary antibodies and was highly time-consuming due to the necessity of antibodies penetrating the brain tissue over large distances (Renier et al., 2014; Susaki et al., 2020). Thus, these protocols were not ready for application in neurological disease models, at least not in larger scales. Using cleared brains, 3D immunohistochemistry was performed in small tissue samples or samples obtained from developing mice (Renier et al., 2014). Due to the small size of tissue samples, the antibodies were able to penetrate the tissue in the latter studies, which was compatible with the tissue clearing and evaluation of anatomical fine structures (Hama et al., 2015). Using a limited set of antibodies exposed over long incubation times, LSFM was recently combined with immunohistochemistry in adult mice (Kirst et al., 2020). The strength of this technique is the visualization of immunohistochemical tissue responses in 3D. Its weakness is the huge effort of tissue processing, including long staining, scanning, data processing, and evaluation times. The utility of these procedures in ischemic brain tissue remains to be evaluated. Analyzing the microvascular responses after transient focal cerebral ischemia requires matching observations in different brain regions with immunohistochemical changes. Reverse clearing followed by brain sectioning and immunohistochemistry provides solutions for this need. LSFM and immunohistochemistry studies can now be combined in the same brains.

The here-presented protocol is ready-to-use in focal cerebral ischemia studies. Infarct and edema size, neuronal densities, leukocyte infiltration, and glia reactivity were very similar in brain sections obtained from brains subjected to reverse clearing and rehydration and PFA-fixed native brains. In a previous study on gingiva samples that were dehydrated with methanol and cleared with benzyl benzoate/benzyl alcohol, rehydration with ascending methanol concentrations enabled hematoxylin, periodic acid Schiff and Giemsa stainings using cryostat sections (Azaripour et al., 2018). However, this protocol has not been systematically compared to samples in which dehydration and clearing had been omitted. Reverse clearing followed by immunohistochemistry will contribute towards decreasing animal numbers in stroke studies, as requested by the Animal Research: Reporting of *in vivo* Experiments ARRIVE guidelines (Kilkenny et al., 2010). This protocol offers itself for use in other neurological disease models, in which reverse clearing and immunohistochemistry will similarly be feasible.

## Data Availability Statement

The original contributions presented in the study are included in the article, further inquiries can be directed to the corresponding author.

## Ethics Statement

The animal study was reviewed and approved by the Landesamt für Natur, Umwelt und Verbraucherschutz, North Rhine Westphalia.

## Author Contributions

AMY, NH, and DMH designed the study. AMY and NH performed the animal experiments. AMY, NH, SS, KW, and YQ performed the brain clearing and reverse clearing experiments. AMY, NH, SS, and KW performed the immunohistochemistries. AMY, NH, MG, and DMH wrote the draft, and all authors revised it. All authors contributed to the article and approved the submitted version.

## Conflict of Interest

The authors declare that the research was conducted in the absence of any commercial or financial relationships that could be construed as a potential conflict of interest.

## Footnotes

^1^http://www.mbl.org/atlas232/atlas232_frame.html

^2^www.vmtk.org
